# Editorial: GABAergic circuits in health and disease

**DOI:** 10.3389/fncir.2023.1322193

**Published:** 2023-10-31

**Authors:** Lisa Topolnik, Graziella Di Cristo, Elsa Rossignol

**Affiliations:** ^1^Department of Biochemistry, Microbiology and Bioinformatics, Laval University, Québec, QC, Canada; ^2^Neurosciences Department, CHU Sainte Justine Research Center, University of Montreal, Montreal, QC, Canada

**Keywords:** GABA, inhibition, interneuron, learning, disorder, epilepsy, PTSD, autism

The intricate web of GABAergic circuits in the brain has long captivated neuroscientists, serving as the backbone of inhibitory control that delicately balances neural excitation. In this Research Topic of articles, we delve into the multifaceted contributions of GABAergic circuits to both the maintenance of cognitive health and the genesis of neurological disorders. A consortium of research teams has explored various aspects of GABAergic inhibition, unraveling novel insights that collectively broaden our understanding of the inhibitory interneuron function and dysfunction. Their groundbreaking work sheds light on how inhibitory circuits impact diverse aspects of cortical information processing, spanning circuit assembly, motor learning, episodic memory formation, epileptiform activities, and fear-related disorders.

Toudji et al. provide a comprehensive overview of the molecular and cellular mechanisms governing the migration of GABAergic interneurons from the ventral telencephalon to their integration within cortical circuits. They delve into the various extrinsic guidance cues, critical interactions with blood vessels, and intrinsic cellular mechanisms that remodel the cytoskeleton to facilitate cellular locomotion in response to these cues. They emphasize how disorders of interneuron migration underlie a subset of genetically determined neurodevelopmental disorders and how cell-based therapies may be envisioned as future therapeutic strategies.

Lee et al. turn their focus to the primary motor cortex, dissecting the role of vasoactive intestinal peptide-expressing (VIP) inhibitory interneurons in motor learning. They reveal how VIP interneurons process diverse inputs, including reward-related information, to orchestrate local circuit plasticity and facilitate reward-based motor learning.

Fossati et al. delve deep into the anterior cingulate cortex, elucidating the response dynamics of cortical layer 1 (L1) interneurons during fear memory processing. Their findings suggest that different subpopulations of L1 interneurons may exert distinct functions in regulating fear learning and memory, offering critical insights into microcircuit organization. This concept is further expanded by Singh and Topolnik, who zoom in on the interplay between excitatory and inhibitory neurons in fear processing circuits across the prefrontal cortex, amygdala, and hippocampus. They highlight the region-specific roles of parvalbumin-, somatostatin-, and VIP-expressing interneurons in fear memory acquisition and related disorders.

Hernández-Frausto et al. provide a comprehensive overview of the multifaceted roles of hippocampal CA1 GABAergic circuits, from shaping neural computations to orchestrating network activity and memory formation. They emphasize the relevance of interneuron dysfunction in the early stages of Alzheimer's disease.

Sun et al. provide novel insights on the biophysical mechanisms by which hippocampal oriens-lacunosum/moleculare (OLM) cells exhibit theta frequency spiking resonance, a feature critical to their ability to modulate hippocampal theta rhythms. By creating a simplified single compartment computational model that preserves the biophysical fidelity of hippocampal rhythm generation, Sun et al. reveal that the combination of hyperpolarization-activated cation and muscarinic type potassium currents specifically enable OLM cells to exhibit theta frequency spiking resonance. They also demonstrate the utility of their novel model in estimating various conductance parameters in real-time from *in vitro* data, providing novel avenues to explore hippocampal rhythm generation.

Piskorowski and Chevaleyre provide an overview of the unique properties of inhibitory transmission and synaptic plasticity in the CA2 region of the hippocampus. They discuss how inhibitory circuit dysfunctions in this region may contribute to deficits in social recognition memory in different neurological and psychiatric disorders, including multiple sclerosis, autism spectrum disorders, Alzheimer's disease, epilepsy, schizophrenia, and 22q11.2 deletion syndrome.

Avoli et al. discuss the paradoxical role of GABAergic signaling in epileptogenesis, uncovering its unexpected involvement in generating interictal discharges and initiating focal seizures. They offer hope for novel pharmacological treatments in focal epileptic disorders like mesial temporal lobe epilepsy.

Dharavath et al. explore how GABAergic signaling is disrupted in alcohol use disorder, including during withdrawal, and provide insights into novel therapeutic avenues aimed at reversing these circuit deficits.

Finally, Gosgnach provides an extensive overview of the roles of different types of GABAErgic interneurons in the regulation of locomotion at the level of the spinal cord central pattern generators, with a focus on the mechanisms regulating left-right and flexor-extensor alternation. These mechanistic insights inform the development of novel strategies to enhance the recovery of motor function following spinal cord injuries.

As we navigate this collective journey through the labyrinth of GABAergic circuits, we gain valuable insights into their pivotal roles in health and disease, painting a rich portrait of the brain's intricate balance between excitation and inhibition. These novel insights not only expand our theoretical understanding but also hold promise for the development of innovative therapeutic interventions in the realm of neurology and psychiatry.

## Author contributions

LT: Writing – original draft, Writing – review & editing. GD: Writing – original draft, Writing – review & editing. ER: Writing – original draft, Writing – review & editing.

